# Modeling Immune Checkpoint Inhibitor Efficacy in Syngeneic Mouse Tumors in an Ex Vivo Immuno-Oncology Dynamic Environment

**DOI:** 10.3390/ijms21186478

**Published:** 2020-09-04

**Authors:** Daniel T. Doty, Julia Schueler, Vienna L. Mott, Cassie M. Bryan, Nathan F. Moore, John C. Ho, Jeffrey T. Borenstein

**Affiliations:** 1 Draper, Bioengineering Division, Cambridge, MA 02139, USA; monohedron@gmail.com (D.T.D.); vmott@draper.com (V.L.M.); cbryan@draper.com (C.M.B.); moor8565@gmail.com (N.F.M.); 2 Charles River Research Services Germany GmbH, 79108 Freiburg, Germany; Julia.Schueler@crl.com; 3 Charles River Laboratories, Wilmington, MA 01887, USA; John.Ho@crl.com

**Keywords:** immune checkpoint blockade, cancer, tumor microenvironment, lymphocytes, syngeneic models, microfluidics

## Abstract

The immune checkpoint blockade represents a revolution in cancer therapy, with the potential to increase survival for many patients for whom current treatments are not effective. However, response rates to current immune checkpoint inhibitors vary widely between patients and different types of cancer, and the mechanisms underlying these varied responses are poorly understood. Insights into the antitumor activities of checkpoint inhibitors are often obtained using syngeneic mouse models, which provide an in vivo preclinical basis for predicting efficacy in human clinical trials. Efforts to establish in vitro syngeneic mouse equivalents, which could increase throughput and permit real-time evaluation of lymphocyte infiltration and tumor killing, have been hampered by difficulties in recapitulating the tumor microenvironment in laboratory systems. Here, we describe a multiplex in vitro system that overcomes many of the deficiencies seen in current static histocultures, which we applied to the evaluation of checkpoint blockade in tumors derived from syngeneic mouse models. Our system enables both precision-controlled perfusion across biopsied tumor fragments and the introduction of checkpoint-inhibited tumor-infiltrating lymphocytes in a single experiment. Through real-time high-resolution confocal imaging and analytics, we demonstrated excellent correlations between in vivo syngeneic mouse and in vitro tumor biopsy responses to checkpoint inhibitors, suggesting the use of this platform for higher throughput evaluation of checkpoint efficacy as a tool for drug development.

## 1. Introduction

The emergence of monoclonal antibodies that target immune checkpoint pathways is one of the most promising developments in the recent history of cancer treatment, with extraordinary clinical responses observed for particular groups of patients and specific types of cancers. A pioneering discovery in medicine was the realization that tumors often evade attack by the immune system by dysregulating the normal function of immune checkpoint pathways, and that the use of specific immune checkpoint inhibitors (ICIs) can effectively block these mechanisms of immune resistance [1,2,3]. Immune checkpoint inhibitors target specific pathways such as CTLA4 and PD-1, and have shown remarkable success against melanoma [4], non-small-cell lung cancer [5], and Hodgkin’s lymphoma [6], with mixed results in several other cancers. Since their approval in 2014, two immune checkpoint inhibitors have vaulted into the ten top-selling prescription drugs in the world, and approvals for new drugs and applications to additional types of cancer continue to mount. In spite of these dramatic successes, further progress is limited by additional resistance pathways and the potential need for involvement of multiple checkpoint inhibitors. Exploration of these pathways and mechanisms would benefit from preclinical models with higher throughput; systems that can shed light on underlying mechanisms of resistance, response, and off-target effects; and technologies capable of directly evaluating human tumor tissue. These requirements have spurred efforts toward the development of model systems that recapitulate key aspects of the tumor microenvironment and that can be used to screen responses to emerging ICIs and combination therapies, which will be critical to future advances in immunotherapy [7].

Models of the tumor microenvironment have been under development for more than a decade [8,9], leveraging early pioneering work in microfluidics [10,11,12] and, more recently, microphysiological systems [13]. Many of these developments involve two-dimensional culturing of cancer cell lines, suspended spheroid systems, and organoids cultured in extracellular matrix [14] for the purposes of drug screening [15,16] and elucidating the mechanisms involved in the immune checkpoint blockade [17,18]. However, these models do not faithfully recapitulate the in vivo tumor microenvironment due to alterations in the cellular and matrix composition that may limit their predictive power compared to studies on unmodified tumor fragments [19]. Microfluidic platforms for tumor slices [20,21,22,23] and bioprinting technologies have also emerged [24], as have systems that model angiogenesis, metastasis, extravasation, and other phenomena [25,26,27,28,29,30,31,32,33]. While these systems have shown utility in modeling chemotherapeutic responses and immune checkpoint behavior, most are non-perfused systems that do not capture the dynamics of lymphocyte migration and drug transport [34], and their study is often of limited duration due to a rapid decline in viability beyond 24–72 h [35,36]. An urgent need remains for engineered platforms capable of supporting viable tumor fragments over a longer time frame, accommodating dynamic interactions between lymphocytes and tumors, and enabling real-time imaging in order to quantify tumor killing and lymphocyte infiltration in response to ICI treatments.

In this study, we report on the application of a perfused microfluidic platform technology, termed EVIDENT (ex vivo immuno-oncology dynamic environment for tumor biopsies), to assess the efficacy of two classes of ICIs against three syngeneic mouse tumor models exposed to flowing drug-treated lymphocytes. The system has been previously reported and shown to recapitulate tumor killing induced by anti-PD-1 treatment of tumor-infiltrating lymphocytes (TILs) with the known responder MC38 syngeneic mouse model [37]. Dynamic perfusion of unmodified tumor fragments permits the preservation of viability over periods of 7 days or more, and enables interactions between flowing TILs and spatially entrained tumor tissues. The system is constructed using materials chosen to minimize the drug adsorption phenomena associated with commonly used microfluidic substrates such as poly(dimethylsiloxane) [38], while still providing a high degree of optical transparency for clear image quality [39]. Further, mechanisms of TIL infiltration, migration, and lymphocyte-mediated killing within tumor fragments, which cannot be observed directly in animal studies, are readily accessible in real time during the course of an EVIDENT experiment. These advantages render the EVIDENT system a powerful tool for increasing the efficiency of checkpoint blockade studies, reducing the number of animals required to perform such investigations, and providing mechanistic insights into the key immune cell–tumor interactions.

## 2. Results

### 2.1. Device Construction and Syngeneic Tumors

**Figure 1:**
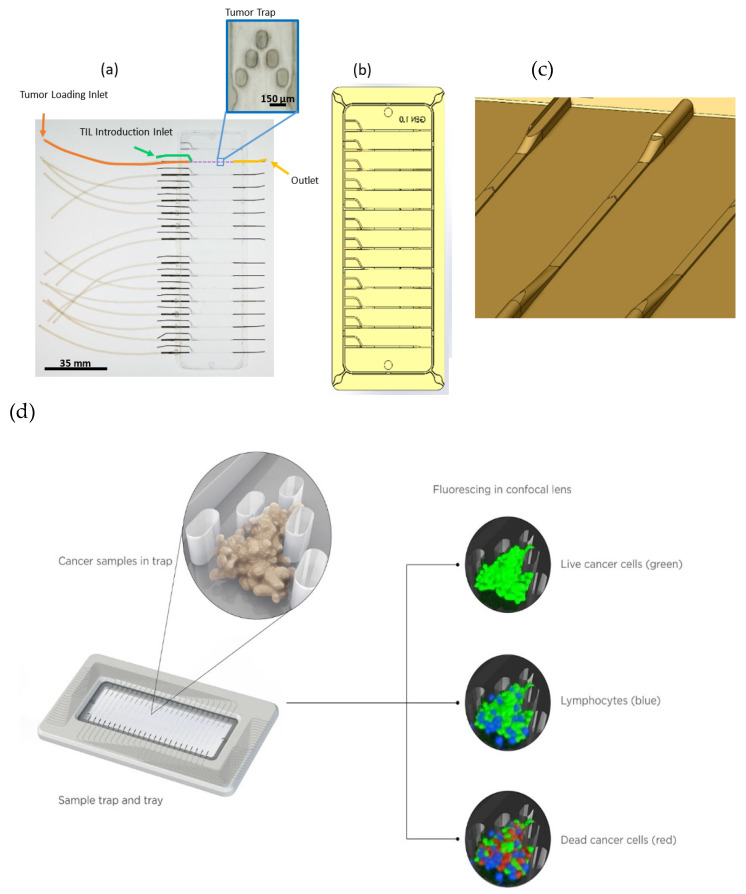
(**a**) Photograph of a top view of the microfluidic device. This device comprises 16 independent channels from two joined partial 12 channel ex vivo immuno-oncology dynamic environment for tumor biopsies (EVIDENT) chips, with two inlet lines—one for tumor loading (orange) and one for TIL introduction (green)—at the left and one outlet line (yellow) on the right corresponding to each channel. The inlet channels are for tumor loading (orange) and for tumor-infiltrating lymphocyte (TIL) introduction (green). Each channel has a tumor trap (blue, inset) located roughly 2/3 of the way from the inlet to the outlet. (**b**) Computer-aided design (CAD) drawing of a single 12 channel chip from a top view. (**c**) Schematic showing the features on the master mold used to emboss the microfluidic path from the inlet—a larger-bore channel narrowing down to the trap region of the channel in the foreground, and then passing through the five post tumor trap region and through the outlet channel at the rear of the drawing. (**d**) Operational schematic that illustrates the processes associated with the images in Figure 2, Figure 3 and Figure 4. The combined device (at left) contains five post traps into which tumor fragments are loaded (top left). At right, CellTracker Green staining of the fragment at the outset indicates live cells in the tumor sample, while lymphocytes are stained with CellTracker Red (blue color), and finally the continuous infusion of annexin V-APC stains dead cells (bottom right). As described in the text, the annexin V-APC stains all dead cells, not just cancer cells.

**Figure 2:**
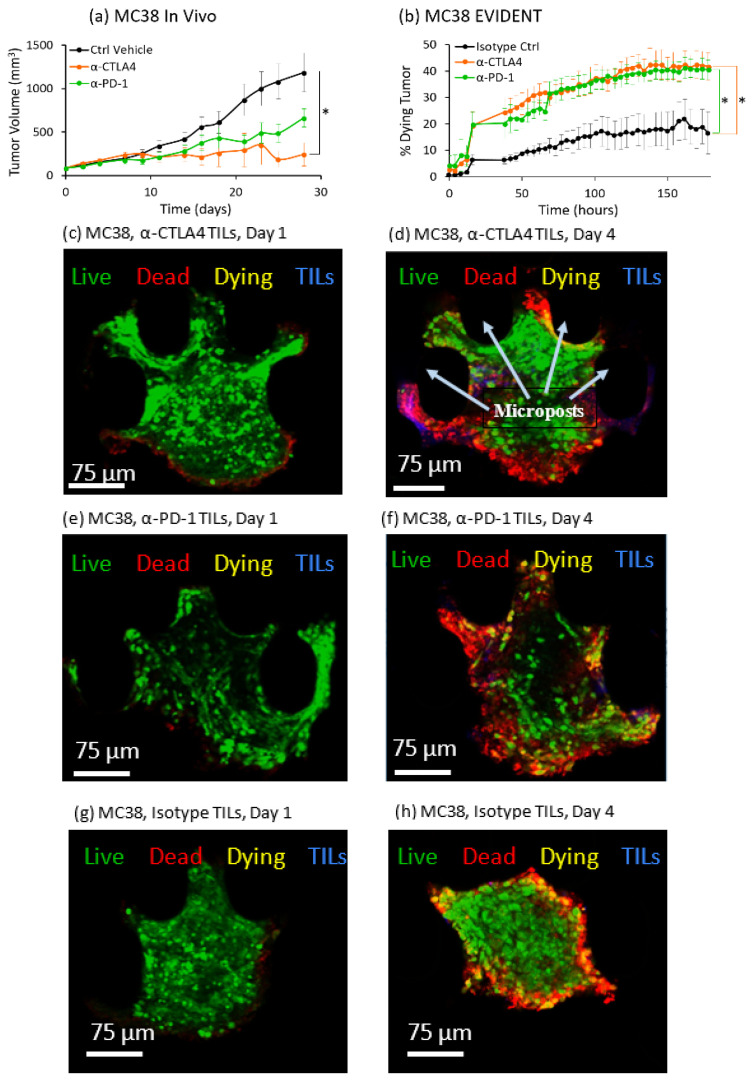
In vivo/in vitro correlation (IVIVC) for MC38 tumors in mice and the EVIDENT system. (**a**) In vivo response of MC38 tumors in mice to α-PD-1 (green), α-CTLA4 (orange), and a vehicle control (black) as a function of tumor growth over time. MC38 tumors showed a strong response to both α-PD-1 (green) and α-CTLA4 (orange), as demonstrated by the delay in tumor growth when treatment was delivered to the mouse on Day 0. Ctrl group: *n* = 8 (days 0–14), 7 (16–25), 6 (28); α-CTLA4 group: *n* = 8 (days 0–9), 7 (11–23), 6 (25–28); α-PD-1 group: *n* = 8 (days 0–7), 6 (9–14), 5 (16–18), 4 (21–23), 2 (25–28). Error bars indicate standard error. * *p* < 0.05 by Kruskal–Wallis test. (**b**) MC38 tumor response in the EVIDENT in vitro system under continuous perfusion of TILs pretreated with α-PD-1 (green), α-CTLA4 (orange), or an isotype control (black) as a function of the percentage of dying tumor tissue over time. There was an increase in the percentage of dying tissue for MC38 tumors treated with either checkpoint inhibitor. *n* = 3/group for all time points. Error bars indicate standard error. * *p* < 0.05 by Kruskal–Wallis test. (**c**–**f**) High-resolution confocal images selected from z-stack of images for MC38 tested in the EVIDENT system. Green = live (CellTracker Green), red = dead (annexin V-APC (allophycocyanin)), yellow = dying (co-localization of CellTracker Green and annexin V-APC), blue = TILs (CellTracker Red). (**c**) MC38 tumor at the start of circulation of α-CTLA4-treated TILs on Day 1. (**d**) MC38 tumor with continuous exposure to flowing α-CTLA4-treated TILs on Day 4. Note that there was extensive death within the core of the tumor, and there was extensive TIL infiltration within the fragment as well. (**e**) MC38 tumor at the start of circulation of α-PD-1-treated TILs on Day 1. (**f**) MC38 tumor with continuous exposure to flowing α-PD-1-treated TILs on Day 4, with cell death visible through the core of the fragment. Microposts are marked in 2d as an exemplary notation (black ovals) to clarify fragment position within the trap. (**g**) MC38 tumor fragment at the start of circulation of isotype-treated TILs on Day 1. (**h**) MC38 tumor with continuous exposure to flowing isotype-treated TILs on Day 4; note that the only death was around the perimeter of the fragment and no TILs were visible within the fragment.

**Figure 3:**
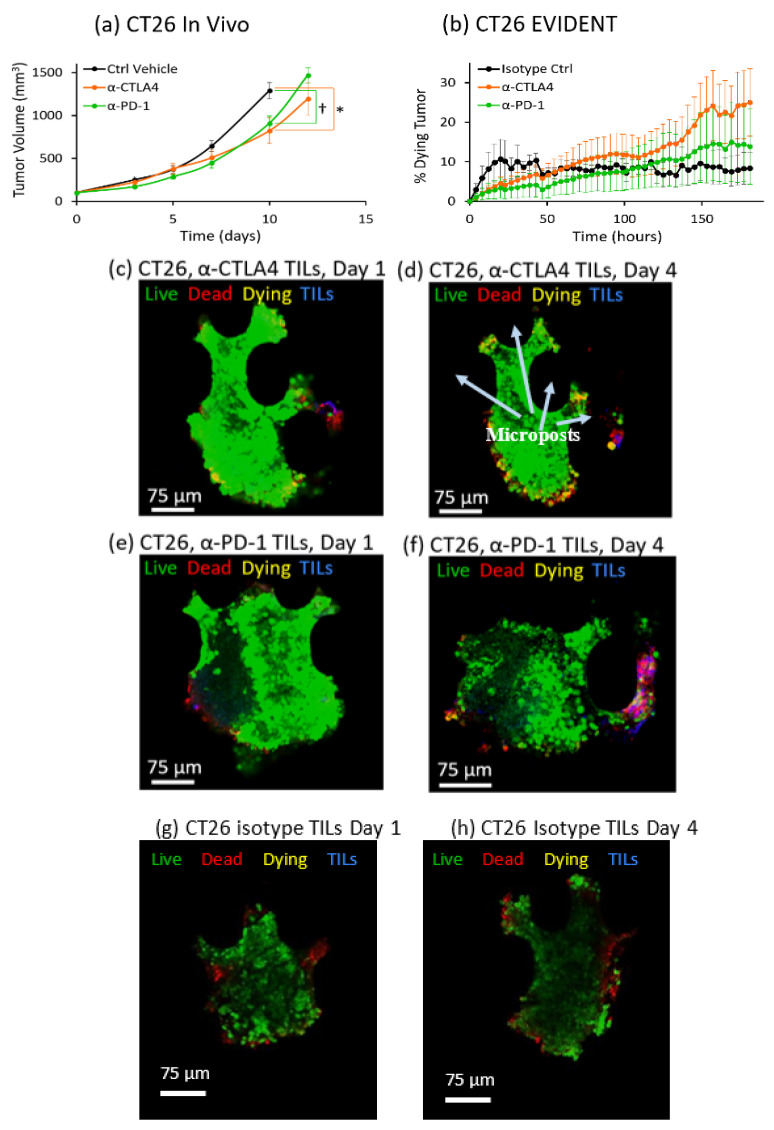
In vivo/in vitro correlation (IVIVC) for CT26 tumors in mice and the EVIDENT system. (**a**) In vivo response of CT26 tumors in mice to α-PD-1 (green), α-CTLA4 (orange), and a vehicle control (black) as a function of tumor growth over time. Tumors in CT26 mice treated with α-PD-1 (green) or α-CTLA4 (orange) had a similar delay in tumor growth compared to the vehicle control (black). *n* = 10/group for all time points. Error bars indicate standard error. * *p* < 0.05, † *p* < 0.01 by Kruskal–Wallis test. (**b**) CT26 tumor response in the EVIDENT in vitro system under continuous perfusion of TILs pretreated with α-PD-1 (green), α-CTLA4 (orange), or an isotype control (black) as a function of the percentage of dying tumor tissue over time. CT26 tumors in the EVIDENT in vitro system showed a small but not significant (*p* > 0.05 by Kruskal–Wallis test) decrease in the rate of dying tissue when perfused with TILs treated with either α-PD-1 (green) or α-CTLA4 (orange) compared to the isotype control (black). *n* = 3/group for all time points. Error bars indicate standard error. (**c**–**f**) High-resolution confocal images selected from z-stack of images for CT26 tested in the EVIDENT system. Green = live (CellTracker Green), red = dead (annexin V-APC), yellow = dying (co-localization of CellTracker Green and annexin V-APC), blue = TILs (CellTracker Red), and magenta = dying TILs (co-localization of CellTracker Red and annexin V-APC). (**c**) CT26 tumor on Day 1 of circulation with α-CTLA4-treated TILs. (**d**) CT26 tumor after 4 days of perfusion with α-CTLA4-treated TILs. (**e**) CT26 tumor on Day 1 of circulation with α-PD-1-treated TILs. (**f**) CT26 tumor after 4 days of perfusion with α-PD-1-treated TILs. Microposts are marked in 3d as an exemplary notation (black ovals) to clarify fragment position within the trap. (**g**) CT26 tumor fragment on Day 1 of circulation with isotype-control-treated TILs, and (**h**) CT26 tumor fragment on Day 4 after continuous exposure to isotype-treated TILs. Note that there was some extrusion-like modulation of the tumor fragment at Day 4 relative to Day 1 as a result of exposure to the flow stream.

**Figure 4:**
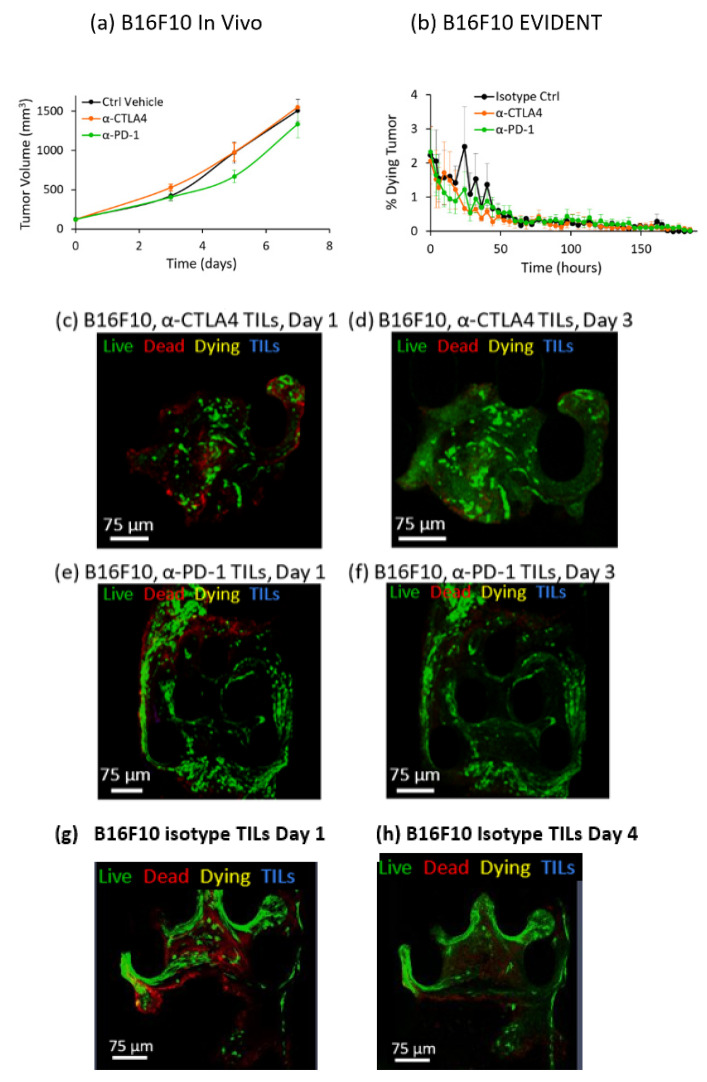
In vivo/in vitro correlation (IVIVC) for B16F10 tumors in mice and the EVIDENT system. (**a**) In vivo response of B16F10 tumors in mice to α-PD-1 (green), α-CTLA4 (orange), and a vehicle control (black) as a function of tumor growth over time. B16F10 mice showed no difference (*p* > 0.05 by Kruskal–Wallis test) in tumor growth following treatment with either checkpoint inhibitor compared to the vehicle control. Ctrl group: *n* = 8 (days 0–3), 7 (5), 2 (7); α-CTLA4 group: *n* = 8 (day 0), 7 (3), 5 (5), 1 (7); α-PD-1 group: *n* = 8 (days 0–3), 7 (5), 3 (7). Error bars indicate standard error. (**b**) B16F10 tumor response in the EVIDENT in vitro system under continuous perfusion of TILs pretreated with α-PD-1 (green), α-CTLA4 (orange), or an isotype control (black) as a function of the percentage of dying tumor over time. B16F10 tumors also showed no difference (*p* > 0.05 by Kruskal–Wallis test) in the percentage of dying tissue over time when perfused with TILs pretreated with either checkpoint inhibitor compared to those treated with the isotype control. *n* = 3/group for all time points. Error bars indicate standard error. (**c**–**h**) High-resolution confocal images selected from z-stack of images for B16F10 tested in the EVIDENT system. Green = live (CellTracker Green), red = dead (annexin V-APC), yellow = dying (co-localization of CellTracker Green and annexin V-APC), blue = TILs (CellTracker Red), magenta = dying TILs (co-localization of CellTracker Red and annexin V-APC). (**c**) B16F10 tumor at the start of circulation of α-CTLA4-treated TILs on Day 1. (**d**) B16F10 tumor with continuous exposure of fragment to flowing α-CTLA4-treated TILs on Day 4. (**e**) B16F10 tumor at the start of circulation of α-PD-1-treated TILs on Day 1. (**f**) B16F10 tumor with continuous exposure of fragment to flowing α-PD-1-treated TILs on Day 4. (**g**) B16 F10 tumor at the start of circulation of isotype control treated TILs on Day 1 and (**h**) on Day 4.

Microfluidic devices used for these in vitro studies were expanded relative to those reported in earlier studies [37]. Two 12 channel devices were cut and combined to create a single 16 channel device, as shown in Figure 1a, where the TIL and medium introduction inlet is shown in green, connected to one of two inlet ports for each of the 16 channels. The corresponding tumor introduction port is shown in orange, connected to the other inlet port. The inset shows an enlarged view of the five post tumor trap positioned approximately 2/3 of the way down each channel from the inlet port toward the outlet port. In Figure 1b, a CAD (computer-aided design) drawing of one of the original 12 channel chips is shown for clarity, indicating each of the inlet/outlet port features. Figure 1c shows a schematic that highlights the flow path through the channel and the tumor trap for each channel, where the features on the inverse master mold used to emboss the microfluidic device are illustrated. Here, the medium and TIL introduction channel narrows down toward the region containing the tumor trap and then expands again toward the outlet. Finally, in Figure 1d, concept drawings showing the operation of the device are provided, where the device is shown at the lower left along with an inset that illustrates an example tumor fragment captured within the five post trap. On the right, illustrations of the fragments in the trap representing the images that are presented in Figure 2, Figure 3 and Figure 4 are provided, showing the initial loading of a fragment in a trap exposed to CellTracker Green, which stains live cells. The image below shows a fragment with lymphocytes that have entered and migrated within the fragment marked in blue (stained with CellTracker Red as described in the Materials and Methods), and at the bottom right, dead cells in the fragment are shown stained with annexin V-APC, which was continuously flowed into the medium. The fragments used in this study were either MC38, CT26, or B16F10 syngeneic mouse lines, chosen for use in this study because of their frequent use in ICI studies and their well-characterized responses to ICI therapeutics (Supplementary Figure S1).

### 2.2. MC38 Syngeneic Mouse Tumors

The abovementioned in vitro response plots were then compared on a qualitative basis to the checkpoint inhibitor response observed in vivo in a mouse model. Mice were given intraperitoneal injections of either α-CTLA4 or α-PD-1, or sham injections as described in the Materials and Methods. The tumor volumes were tracked and the experiment stopped after the volume reached 1500 mm^3^ in each mouse. The in vivo data tracked tumor volume as a function of time, while in vitro results tracked tumor death over time. Therefore, for a responsive tumor, a strong in vitro/in vivo correlation (IVIVC) would be demonstrated by an increase in tumor death in the EVIDENT system and deterrence of tumor growth in mice. In contrast, a strong IVIVC for a nonresponsive tumor would have low levels of dying tumor in the EVIDENT system, while treated mice would have similar tumor growth levels to those treated with control compounds.

The MC38 syngeneic mouse tumor model has been demonstrated by multiple labs to respond to both α-CTLA4 and α-PD-1 treatments in vivo [40,41]. Our independent in vivo experiments were able to replicate this response in mouse studies, and both treatments strongly delayed the growth of MC38 tumors in the mice tested. Mice treated with α-CTLA4 had an 80% reduction in tumor volume and those treated with α-PD-1 had a 45% reduction in tumor volume on average on Day 28 compared to mice that received a vehicle control (Figure 2a). The EVIDENT system mirrored these in vivo findings. Both treatments resulted in a higher percentage of tumor fragment death than of the isotype control fragments, with α-CTLA4 yielding a 2.5-fold increase and α-PD-1 yielding a 2.4-fold increase at the last time point taken (Figure 2b). Figure 2c shows a z-slice image of an MC38 tumor fragment after 1 day of perfusion with TILs pretreated with α-CTLA4. Viability was high on Day 1, with little presence of annexin V-APC marking tumor death. By Day 4, a significant region within the MC38 fragment had been invaded by lymphocytes (blue) and cell death was prevalent (red) (Figure 2d). A similar increase in dead (red) and dying (yellow) tissue resulting from the increase in TIL retention (blue) was also seen for MC38 tumors perfused with α-PD-1-treated TILs in the microfluidic system from Day 1 (Figure 2e) to Day 4 (Figure 2f). Figure 2g shows an image of an MC38 tumor fragment on Day 1, exposed to TILs treated with isotype control. At Day 4, following continuous exposure to flowing TILs treated with isotype control, the only visible death signal was seen around the perimeter of the fragment (Figure 2h). While these studies used very different measurements and time scales, these two approaches demonstrated a strong IVIVC based on the delayed tumor growth in vivo and the increased rate of death in EVIDENT as proxies for response to treatment. Appearance of a magenta color, particularly in Figure 2d, showed the overlap of CellTracker Red and annexin V-APC, indicating areas of concentrated TIL death.

The opportunity to track TIL migration within tumor fragments is another exciting aspect of the EVIDENT system. In Supplementary Figure S2, we show time-lapse images taken over the course of exposure of one of the MC38 tumor fragments to circulating TILs that were exposed to α-PD-1 prior to their introduction into the system. These time-lapse images, taken every 3 h over the course of Day 2, showed TIL penetration and migration, particularly at the left edge of the fragment, with increasing death in those regions. Additional images taken at the end of Day 3 and Day 4 are provided to show the reduction in live signal (green), associated with cell death but also with fading of the CellTracker green signal, along with an increase in annexin V-APC signal as more of the fragment tissue died.

### 2.3. CT26 Syngeneic Mouse Tumors

The CT26 syngeneic murine tumor model has previously been shown by multiple sources to have a low-to-moderate response to checkpoint inhibitors [40,41]. The mice in this study responded moderately both to α-CTLA4, with a 36% decrease in average tumor volume, and to α-PD-1, with a 30% decrease in tumor volume 10 days post-treatment (Figure 3a). Over time, the in vivo mouse studies demonstrated that the response to α-CTLA4 became stronger than the response to α-PD-1, with a divergence between the average responses relative to the mouse control. This difference caused the tumor growth to begin to slow markedly in response to α-CTLA4 over extended periods, with a decreasing slope in tumor volume versus time, while the α-PD-1-treated mice showed a slope in tumor growth versus time relatively similar to that seen for control-treated mice, with a gap or delay in tumor growth. This poorer response to α-PD-1 distinguished the CT26 model from MC38, and therefore provides a different reference for the EVIDENT system to be tested against.

Direct comparison between the in vivo results and those obtained using the EVIDENT model showed that the EVIDENT system also provided a level of response for CT26 distinct from the previously discussed MC38 model. In EVIDENT, there was a 3.0-fold average increase in percentage dying tissue for tumors perfused with α-CTLA4-treated TILs and only a 0.6-fold average increase for those perfused with α-PD-1-treated TILs compared to tumors perfused with isotype-control-treated TILs (Figure 3b). These factors were calculated at the final time point in Figure 3b, where the baseline (isotype control) dying tumor level was 8%, the corresponding α-CTLA4 level was 25% (approximately 3-fold increase), and the α-PD-1 dying tumor level was 13%, a roughly 0.6-fold increase. This difference in response to the two checkpoint inhibitors in EVIDENT was consistent with the mouse studies, and distinguished the EVIDENT results for CT26 from those obtained for MC38. Interestingly, these differences observed in vitro were not found to be statistically significant in a Kruskal–Wallis test, in agreement with previous results that CT26 tumors are generally less responsive to checkpoint inhibitors. A small increase in dying tumor tissue was seen in the confocal images of CT26 tumors in the EVIDENT device perfused with α-CTLA4-treated TILs or α-PD-1-treated TILs on Day 1 (Figure 3c,e) compared to Day 4 (Figure 3d,f). Interestingly, both in vivo and in vitro, treatment groups seemed to only begin to diverge at the later time points, highlighting the advantages of in vitro systems that maintain viable fragments for longer periods to permit later mechanisms to be observed. Images of CT26 tumors exposed to TILs treated with isotype control are shown for Day 1 (Figure 3g) and Day 4 (Figure 3h), with minimal evidence of TIL-mediated death over the course of the study.

### 2.4. B16F10 Syngeneic Mouse Tumors

B16F10 syngeneic tumors do not historically respond appreciably, on average, to either α-PD-1 or α-CTLA4 treatment in vivo [42]. We also found that neither treatment significantly affected the growth of B16F10 tumors compared to the vehicle injection in mice (*p* > 0.05 in Kruskal–Wallis test) (Figure 4a). The in vivo data showed that the α-CTLA4 treatment resulted in an indistinguishable change in tumor growth from that seen in control-treated mice. Treatment with α-PD-1 caused a very slight drop in tumor growth, but well within the variation seen between animals. Therefore, the B16F10 model serves as a useful baseline for the EVIDENT system, in that neither ICI drug treatment produced a significant response in tumor growth/tumor killing.

In EVIDENT, all treatment groups had a modest fraction of dying tissue on Day 0, likely due to tissue damage from tumor fragmentation and device loading (Figure 4b). Note that the axis in this figure is greatly expanded, indicating that the level of tissue death was very small relative to the other studies. However, the percentage of dying tissue was relatively stable from this starting value over the course of the experiment, and there was negligible new cell death. In the case of the EVIDENT system, there was in fact a slight drop in the level of measured tumor death over time, but with the expanded scale in this chart, this change was marginal at best, and therefore the overall EVIDENT result can be summarized as indicating negligible TIL-mediated tumor killing for all of the cases studied. As shown in the example images in Figure 4, B16F10 tumor fragments retained much of their live stains (green in images), and did not gain annexin V-APC staining (red in images) between Days 1 (Figure 4c,e) and 4 (Figure 4d,f), indicating that the viability remained high in the fragments over several days in perfusion flow. Figure 4g,h shows B16F10 tumor fragments exposed to isotype-treated TILs on Day 1 and Day 4, respectively, with no visible effects on either TIL infiltration or death signal.

## 3. Discussion

This study showed for the first time that a dynamic in vitro tumor microenvironment can be utilized to distinguish differences in response between two different ICI therapies, anti-CTLA4 and anti-PD-1, against three different syngeneic mouse lines, MC38, CT26 and B16F10, all well-established models for cancer research [40,41]. Further, the EVIDENT model effectively increases the throughput from a single data point per animal tested to 16 data points obtained from a single chip using fragments derived from a single animal, with the potential for much higher throughput as the microfluidic device is scaled up. The EVIDENT results obtained on the three different syngeneic mouse tumor lines for each of two checkpoint inhibitors and a control compound were contextualized by comparison with in vivo studies using the same tumor models and same treatment compounds. The in vivo studies utilized tumor volume over time as a readout, with monitoring ceasing when the volume reached an endpoint of 1500 mm^3^. Tumor volume was tracked as a function of standard intraperitoneal doses of single anti-PD-1 or anti-CTLA4 checkpoint inhibitors, as well as a control case. Average values for absolute tumor volume were plotted versus time. Increased checkpoint efficacy was seen to suppress tumor growth in these studies, providing a baseline for the evaluation of the EVIDENT in vitro model.

In contrast with the in vivo studies on syngeneic mice, the readout for the in vitro EVIDENT experiments was the percentage of dying tissue in the tumor fragment, as measured by co-localization of live tumor stain and dead stain tracked over time. This metric, the rate at which the tumor fragment is dying, can be evaluated as a function of checkpoint inhibitor treatment for each tumor type. In the EVIDENT studies, checkpoint treatments were applied to TILs originating from sister fragments of the sample under test, as described in detail in the Materials and Methods and in Reference [37]. Once tumors were loaded into the EVIDENT device, no drug was present in the circulating media, only the treated or untreated TILs, so that the response to the drug was driven solely by the action of the compound on TILs prior to their exposure to fragments. Any resident TILs present in the biopsied fragment samples were not exposed to free drug, although there is the potential for a basal level of resident TIL activity against the tumor, as well as the possibility of cross-talk between flowing and resident TILs.

To accurately quantify ICI response in the EVIDENT system, we needed to carefully choose the correct readout that would most closely represent the changes occurring in the tumor fragments in response to exposure to pretreated TILs. The rationale for using co-localization of the CellTracker Green, which is retained in cells with uncompromised membranes, and annexin V-APC, which stains phosphatidylserine on the outer membrane of dead and dying cells, was an attempt to capture cell death directly associated with a drug response. This co-localization was interpreted as indicating that the tumor cell was more likely to be in an early apoptotic “dying” state, as opposed to a “dead” cell that was solely annexin-V-positive, which might have many possible causes. Such causes included initial dead cells present in the as-received sample, cells damaged during fragmentation, and cells at the perimeter of the fragment that were damaged by the flow or proximity to EVIDENT trap features. These and other possible causes of annexin-V-APC-positive cells might result in spurious death signals unrelated to ICI-driven response, and they were eliminated from our analysis by the requirement for co-localization to register as tumor killing in response to ICI treatment of TILs. Using this analysis method, a tumor fragment could be seen to respond to ICI-treated TILs by dying at a greater rate than fragments exposed to isotype-treated TILs.

Results for ICI response in the EVIDENT system were consistent with in vivo mouse studies across three different syngeneic murine tumor models using the same treatment compounds. Comparison of the in vivo and EVIDENT in vitro data required the establishment of a correlation between tumor volume in the mouse and tumor fragment death in the microfluidic system. This was relatively straightforward in a qualitative sense, although strict quantitation was made difficult in part by the relative simplicity of the model and the accelerated timeline seen for small fragments. In essence, the tumor killing seen in the EVIDENT system is a barometer of drug efficacy, just as is the suppression of tumor growth in the in vivo data. An acceleration of the timescale, from several weeks in vivo to several days in EVIDENT is likely due to the small size (approximately 150 μm diameter) of the fragment relative to the mouse tumor (ca. 10 mm diameter), and the fact that TILs were not required to home and infiltrate very deeply into the tumor fragments. The use of two different checkpoint inhibitors permitted assessment of the relative efficacy of each treatment in both applications, and therefore served as an initial validation of the appropriateness and relevance of the model, although additional work is required to more fully validate the EVIDENT platform, as these studies focused on one particular mode of interaction between lymphocytes and tumors. In particular, investigation of the effects of ICI applied directly to perfused fragments in the EVIDENT system may be compared with those observed for ICI-treated TILs interacting with fragments, which would shed light on mechanisms such as TIL exhaustion, drug transport, and lymphocyte migration in the tumor.

One of the greatest challenges facing any in vitro platform technology is tumor fragment variability, and this is also the case with the EVIDENT system. Biopsied fragments could vary in size, shape, or composition. Indeed, because the tumor fragments varied in size, the normalized signal was a more comparable metric than absolute “dying” volume. These factors could also affect, among other things, the number of TILs that encounter the fragment, how the TILs make contact, and various edge effects due to geometric variation. As was the case with the syngeneic mouse lines in the in vivo studies, there were individual tumor fragments that did not behave like the average of their treatment groups. This variability, with only three fragments per group, presented a challenge for statistical analysis of the EVIDENT results. This could be addressed in future versions of EVIDENT via two paths: (1) by making the tumor fragmentation process more consistent, and (2) by increasing the number of fragments tested per condition. The former will require the development of new techniques, while the latter will require a new EVIDENT device design with a greater number of accessible channels. An additional key advance would be the ability to distinguish normal tissue from cancer cells at a high optical resolution, which would remove a major source of variability between fragments under investigation.

Another exciting potential feature of the EVIDENT system that has yet to be fully explored is tumor infiltration tracking. In these experiments, the presence of the labeled TILs were noted merely as an indication that live TILs were reaching the tumor fragments and binding to them. Binding ability is one important step towards TILs destroying tumor cells, but being at the site of the tumor is not necessarily indicative of a robust response. This is clearly demonstrated by the presence of inactive TILs autochthonous to the tumor. The EVIDENT system allows the possibility of reporting x–y–z coordinates for each reported area. With a more sophisticated analysis pipeline that is currently in development, it will be possible to track how far into the tumor fragment the TILs are able to migrate as an additional marker of functionality.

It is important to acknowledge that many questions and challenges remain in modeling immune checkpoint blockade. These early investigations are only the beginning of the development of the complex tools necessary to explore the full range of key immune–tumor interactions involved in the process. For instance, the transition of technologies such as EVIDENT from syngeneic mouse tumor platforms to human tumor samples is quite challenging, due to the highly heterogeneous nature of human tumors and the variable features present in biopsied samples from patients. Additional complexity relates to efforts to capture the complexity of the immune response in a relatively simple system; it remains unclear to what degree response to ICIs is driven by interactions between checkpoint compounds and TILs present in the tumor relative to effects on circulating lymphocytes distant from tumor loci. Recent evidence suggests that the latter represents perhaps the predominant mechanism for ICI response [43], and therefore the next generation of immuno-oncology models may be required to incorporate circulating immune cells in order to accurately represent the process. Toward this end, future studies with the EVIDENT system might incorporate non-TIL immune-cell populations derived from companion blood samples from mice or humans, and may evaluate the tumor-killing response as a function of the numbers of TILs or other immune cells reaching the tumor fragments. Such experiments present challenges due to greater complexity in the immune component of the system and in quantification of the immune cell populations involved. In spite of these and other challenges, this early investigation provides encouraging evidence that rapid assessment of ICI efficacy can be recapitulated ex vivo in a model system as a powerful tool for future drug development and precision medicine.

Results provided by the EVIDENT system and described here may be assessed in reference to alternative in vitro or ex vivo methods for evaluation of the efficacy of chemotherapeutic compounds or ICIs, all of which have the potential to provide higher throughput and more rapid indications of drug response than in vivo studies Promising results have emerged from studies with tumor spheroids, integrated in some cases with flow and immune cells [17], but these face challenges associated with recapitulating the tumor microenvironment. Significant progress has been made toward integrating microfluidic perfusion systems with tumor slices [22]; however, the duration of these studies is limited and challenges with integrating immune components remain. In this context, the EVIDENT system represents a potential path toward high-throughput dynamic models that integrate immune cells and perfusion flow with tumor fragments closely representative of the in vivo microenvironment. Future applications of this system might explore the relative roles in tumor killing of immune cells derived from various sources, comparing the effects of circulating TILs as in this study with naïve T cells obtained from the blood and introduced into the circulation in the EVIDENT system.

Finally, there are broader potential impacts of this technology on the field of immuno-oncology, ranging from the ability to probe mechanistic phenomena at the cellular level in dynamic studies to rapid assessment of combination therapies, and ultimately in the context of precision medicine placed directly in the clinic. Tools such as EVIDENT can bridge the gap between current high-throughput in vitro screens and animal studies to reduce and more precisely target the latter stage in preclinical development. For instance, the EVIDENT study required a much smaller number of mice than would a comparable in vivo study. In clinical applications, the small size of EVIDENT fragments will enable sampling of tumor biopsies at negligible risk for the patient. The benefit, a highly predictive and timely estimation of the most efficacious treatment protocol, has the potential to allow identification of the best suited therapy regimen for the individual patient at a specific stage of the disease.

## 4. Materials and Methods

### 4.1. Device Fabrication

A microfluidic cyclin olefin copolymer (COC) device was designed with an array of posts utilized to trap tumors in the center of the channel while allowing for medium and TIL flow around and over tumor fragments, as described previously [37]. Posts 75 µm in diameter and 150 µm in length were aligned approximately 100 µm apart in a chevron-like pattern. This array was centered roughly two-thirds of the distance along the length of a 25 mm long, 600 µm wide, and 125 µm deep channel. Twelve parallel and independent channels comprised each glass-slide-sized chip, totaling 95 mm in length and 35 mm in width. Each channel consisted of two inlet ports, one for flow of TILs in the medium and the other for initial introduction of the tumor tissue, as well as one outlet port.

Fabrication of the microfluidic device consisted of a precision-patterned aluminum master mold. Conventional machining was utilized to create a negative imprint of the 12 lane channel, post, and port design. The aluminum metal mold was sputter-coated with 1000 A of chrome and 4000 A of gold, and then soaked in a 1 mM solution of hexadecanethiol, which formed a self-assembled monolayer, to improve polymer release. The master mold was used to hot-emboss (Carver Press, Wabash, IN, USA) the patterned COC chip. A stack consisting of the following materials, from bottom to top, was placed in the Carver press: 125 μm Kapton (Fralock, Valencia, CA, USA), 3/32” rubber sheet (McMaster-Carr, Robbinsville, NJ, USA), machined Al master mold, 2 mm thick COC (8007X-10, TOPAS, Polyplastics USA, Florence, KY, USA), 1/32” polished steel plate, 1” thick Al plate. The stacked materials were heated to 100 °C at 4000 lbs of applied pressure for 30 min, followed by a cooling to 45 °C under 4000 lbs of pressure for 5 min. Spillover material was removed from the edges upon release of the COC chip from the master mold.

Fabrication of the microfluidic device included bonding of the COC chip to a 125 µm COC film using a heated lamination process. The COC chip and COC film were placed between 125 μm Kapton sheets and heated to 74 °C at 90 psi for 30 min to seal the bond. After bonding, 21 gauge stainless steel tubing (Component supply, Fort Meade, FL) was inserted into ports and sealed with an adhesive (R-36, Hapco, Inc., Hanover, MA, USA). Next, Tygon^®^ tubing (Cole-Parmer, Vernon Hills, IL, USA), was slipped over the metal for fluidic access.

The microdevice consisted of 12 independent channels on one chip. However, it is important to note the scaling potential of this device. In an attempt to circumvent resource-intensive fabrication methods, a fully functional proof-of-concept device was developed to demonstrate scaling to 16 channels on one chip. An example of such a device is shown in Figure 1, where tubing connections on the left side for linkage of the media and TIL flow to the device tumor traps are shown (green), along with unconnected tubing fittings at the left for each tumor introduction port (orange) and at the right for the downstream channel exit (yellow). The number of channels was limited by the stage of a standard confocal microscope, but could be increased for other applications.

### 4.2. Microfluidic System

The microfluidic device was placed in series with various components to allow for real-time high-resolution confocal imaging of the tumor fragments, as described previously [37]. A single conical vial was used to contain the medium and TILs for a given condition. Replicates were obtained by inserting multiple fluid lines into a particular vial.

Medium flowed from the reservoir to a peristaltic pump (lsmatec c.p. 78001-20, Cole-Parmer, Vernon Hills, IL, USA) through 24” of 30 gauge polytetrafluoroethylene tubing having an inner diameter of 304.8 µm. Before entering the device, medium flowed through a custom-manufactured bubble trap. The bubble traps were arrayed and each consisted of an acrylic block with milled cylindrical cavities. Medium entered through a port on the side of each cavity and exited through the bottom, causing bubbles to buoyantly rise and become trapped, where a venting port allowed for removal of excess gas as needed. The bottom of each cavity was given a conical shape to funnel media and cells toward the exit port.

Twelve inch 30 gauge polyether ether ketone (PEEK) tubing with an inner diameter of 127 µm connected the bubble trap to the device. A further 12” of PEEK tubing guided fluid from each outlet port of the microfluidic device to a common reservoir, open to the atmosphere. The length and inner diameter of the fluid circuit comprising all tubing segments and the flow channels in the device defined a hydraulic resistance which determined the relationship between applied pressure and medium flow rate, and optimization studies resulted in a combination of resistance and pressure leading to specific ranges of flow rate and shear aimed at mimicking tumor interstitial pressures. Of the various elements of the fluidic circuit, the parameters of the outlet high-resistance PEEK tubing lines were predominant in determining flow rate. For these experiments, the length and inner diameter of the PEEK tubes were chosen such that their fluidic resistances resulted in flow rates of approximately 1–2 μL/min in each line. Fluidic outputs of the system were measured to verify flow conditions and were accurate within 10% of the expected value in every channel.

### 4.3. Cell Culture

Mouse cancer cell lines MC38, CT26, and B16F10 were expanded in RPMI 1640 (Gibco, Waltham, MA, USA) supplemented with 10% FCS (ThermoFisher Scientific, Waltham, MA, USA) and 0.05 mg/mL gentamycin (ThermoFisher Scientific, Waltham, MA, USA) in 5% CO_2_ at 37 °C until 70–80% confluence (60% for MC38). Harvesting cells for subcutaneous injection into mice was done using TripleE Express (ThermoFisher Scientific, Waltham, MA, USA).

### 4.4. Animal Experiment

The following specifications for the subcutaneous cell line implantation were applied: B16F10 was injected into B6D2F1 animals using 5 × 10^5^ cells in 100 µL PBS per animal. MC38 was injected into C57BL/6N animals using 1 × 10^6^ cells in 50 µL PBS and 50µL Matrigel per animal. CT26 was injected into Balb/c animals using 3 × 10^5^ cells in 100 µL PBS per animal. For all experiments, 4–6 week old female mice were used (Charles River, Sultzfeld, Germany). Animals were stratified into different treatment arms when median tumor size reached 80 mm^3^. Tumor volume and body weight measurements were performed three times per week. 10 animals per group were stratified into the treatment arms: (a) control vehicle PBS, 5 mL/kg/d twice weekly i.p.; (b) α-mCTLA4 (clone 9H10, Bioxcell, West Lebanon, NH, USA) 5 mg/kg/d twice weekly i.p.; (c) α-PD-1 (clone RPM1-14, Bioxcell, West Lebanon, NH, USA) 5 mg/kg/d twice weekly ip. Individual animals were sacrificed when tumor volume reached 1500 mm^3^ or other termination criteria applied (e.g., ulceration, body weight loss > 20%).

### 4.5. Preparation of Tumor Fragments and TILs for the Ex Vivo Platform

The cell line implantation was performed as described above. Ten animals were implanted per tumor line and individual animals were sacrificed when tumor volume reached 1000 mm^3^. Three tumors were cut into fragments of 60–120 mm^3^ size and fragments were frozen in 10% DMSO and 90% FCS for later introduction into the EVIDENT device. Five tumors were used to generate a single cell suspension and the tumor-infiltrating lymphocytes (TILs) were isolated using the MACS separation system (Miltenyi Biotec, Bergisch Gladbach, Germany) with the mouse CD45 MicroBeads (#130-110-618, Miltenyi Biotec, Bergisch Gladbach, Germany). Isolated TILs were frozen in 10% DMSO and 90% FCS.

Tumor tissue (400–600 mm^3^) was harvested and incubated with the primary anti-mouse antibody: CD3e-FITC (553062; BD Bioscience, San Jose, CA, USA), Ly-6G-PerCp-Cy5.5 (127615; Biolegend, San Diego, CA, USA), CD45-AF700 (103127; Biolegend), CD11b-APC-Cy7 (561039; BD Bioscience), CD4-eF450 (48-0042-82;Thermo Fisher), Ly-6C-BV 605 (563011; BD Bioscience), CD8-BV650 (100741; Biolegend) CD25-PE (553866; BD Bioscience), FoxP3-APC (17-5773-82; eBioscience); LD-AquaZombie (423102; Biolegend), CD11b-APC/Cy7 (561039; BD Bioscience), F4/80-BV421 (123137; Biolegend), CD335-BV605 (137619; Biolegend), CD49b (DX5)-PE (12-5971-82; Thermo Fisher, Waltham, MA, USA), CD206-APC (17-2061-82;Thermo Fisher) or isotype control, and the mean fluorescence intensity was analyzed by flow cytometry. Staining was performed in the presence of CD16/CD32 Abs to block nonspecific staining (rat anti-mouse CD16/CD32(FcγIII/II) receptor IgG2b; #553142 BD Bioscience). Samples were analyzed on an Attune Acoustic Focusing Cytometer NXT (Applied Biosystems) which recorded 50,000 events. Flow cytometry analysis is provided in Supplementary Figure S3.

On Day 1, one tumor was gently thawed in a water bath, removed from its freezing medium, and placed into prewarmed 99% FBS with 1% penicillin/streptomycin (ThermoFisher 10378016). The tumor was then fragmented using a 10 mL syringe with a 25 g needle by pulling cores of the larger tumor segment. Tumor fragments were collected in the syringe and deposited into a 15 mL conical tube. The remaining volume was filled with sterile PBS, and the fragments were centrifuged at 300× *g* for 5 min. The supernatant was aspirated and 5 mL of PBS with 5 μM CellTracker™ Green CMFDA Dye (ThermoFisher C7025) was added to the tube. The pellet was gently agitated to separate fragments, which were then incubated with the dye for 1 h at 4 °C. After staining, the fragments were washed with complete medium (RPMI (ThermoFisher 11835055), 10% heat-inactivated FBS (ThermoFisher 16140071), 1% penicillin/streptomycin (ThermoFisher 15140122), 1% GlutaMAX™ (ThermoFisher 35050079), 10 mM HEPES (4-(2-hydroxyethyl)-1-piperazineethanesulfonic acid) (ThermoFisher 15630080), 55 μM 2-mercaptoethanol (ThermoFisher 21985023)) and transferred to a six well plate. Fragments of appropriate size (150–300 μm in diameter) were transferred to a fresh well containing complete medium for sorting and were then selected for dye brightness and cellularity under the microscope before being carefully loaded into a primed EVIDENT device. Once loaded with tumor fragments, the device was connected to the system, any bubbles were purged from the device, and the tumor introduction ports were capped off. At least three randomly selected channels were assigned to draw from each of three reservoirs to accommodate three conditions. The entire system was incubated at 37 °C with 5% CO_2_ while complete medium with 1:1000 annexin V-APC conjugate (ThermoFisher A35110) was pumped at 2 μL/min to each channel.

TILs were also gently thawed on Day 1 and added to 5 mL prewarmed FBS. TILs were washed and resuspended in complete medium with 100 U/mL mouse IL-2 (Sigma I0523-20UG). TIL concentration was adjusted to 10^6^ cells/mL and incubated with 5 μM CellTracker™ Red CMTPX (ThermoFisher C34552) in a 37 °C incubator for 30 min. The TILs were washed, resuspended in compete medium with 100 U/mL IL-2 at 10^6^ cells/mL, and split equally between three wells of a 24 well plate. Next, 10 μg/mL of each antibody (isotype control, anti PD-1, and anti CTLA4) was added to each well to create the three treatment conditions. The TILs were incubated overnight at 37 °C with 5% CO_2_.

### 4.6. Perfusion Experiment and Image Collection

The overnight incubation allowed the tumor fragments to be perfused and fully stained by the annexin V-APC, and gave the TILs some time to become pretreated at a high dose of antibody. On Day 0, tumors were imaged for baseline death before TILs were introduced into the medium reservoirs at a final concentration of 1.5 × 10^5^ cells/mL and pumped at 2 μL/min. Imaging was begun on the tumor fragments with three channels using a Zeiss LSM780 confocal microscope: live tumor (488 ex., 494–591 em.), TILs (561 ex. 597–632 nm em.), and dead cells (633 ex. 640–750nm em.). Exposure and laser power were set for each image set, with laser power kept below 7% to avoid impacting the cells in device. Z-stacks were taken, capturing the entirety of each tumor fragment in 10 μm slices at 3–4 h intervals. The system was serviced as needed, which involved removing bubbles from the device and refilling reservoirs with complete medium with 100 U/mL mouse IL-2.

The experimental design for each study included at least nine randomized tumor fragments loaded into tumor traps in the EVIDENT chip. These “trapped” tumors were then perfused with media containing TILs removed from sister fragments of the same tumors that were pretreated with an isotype control (IgG), α-CTLA4, or α-PD-1 for 3–7 days. No drug was present in the circulating medium, so that the response to the drug was driven solely by the action of the compound on TILs before their exposure to the fragments, and any resident TILs present in the biopsied fragment samples were not expected to play a role in the drug response in this system.

### 4.7. Image Analysis

Images were analyzed using Zen software for Zeiss. A low intensity threshold was set for each channel of each set of images. These thresholds also applied to co-localized areas of dyes. A minimum area was set to avoid counting noise. The software found discrete areas of positive signal, defined by the input thresholds, and classified them by the channels for which they were positive, e.g., red only vs. red + green. Each of these areas could be sorted by fragment, time point, and class, and areas were binned and added together to obtain cumulative values. Briefly, dying tumor fraction was determined through co-localization of annexin V-APC and CellTracker Green for each pixel, and this was sum divided by the total tumor volume to obtain a normalized fraction of dying tumor at each time point. The percentage of dying tumor was plotted against time, and these results were used to assess the degree of response for each tumor type to an immune checkpoint inhibitor, α-PD-1 or α-CTLA4, or isotype control. Dying TIL populations, connoted by magenta (co-localization of CellTracker Red and annexin V-APC), was observed but not analyzed specifically.

## Supplementary Materials

The following are available online at https://www.mdpi.com/1422-0067/21/18/6478/s1.

## Abbreviations

APC	Allophycocyanin
CAD	Computer-aided design
COC	Cyclic olefin copolymer
CTLA4	Cytotoxic T Lymphocyte Associated Protein 4
DMSO	Dimethylsulfoxide
EVIDENT	Ex vivo immuno-oncology dynamic environment for tumor biopsies
FCS	Fetal calf serum
ICI	Immune checkpoint inhibitor
IVIVC	In vitro/in vivo correlation
PD-1	Programmed Cell Death Protein 1
PEEK	Polyether ether ketone
TILs	Tumor-infiltrating lymphocytes
