# Supplementary material for: Modeling Immune Checkpoint Inhibitor Efficacy in Syngeneic Mouse Tumors in an Ex Vivo Immuno-Oncology Dynamic Environment
Source: Int J Mol Sci. 2020 Sep 4;21(18):6478. doi: 10.3390/ijms21186478 (PMC7555450; doi:10.3390/ijms21186478)
Supplement: Supplementary file 1 [file ijms-21-06478-s001.pdf]

MC38, colon cancer model, chemically induced by DMH in C57Bl/6 mice,
high mutational load (59 TMB/MB)

CT26, colon cancer model, chemically induced by NMU in balb/c mice,
high mutational load (63 TMB/MB)

B16F10, melanoma model, spontaneous formation in C57Bl/6 mice,
low mutational load (29 TMB/MB)

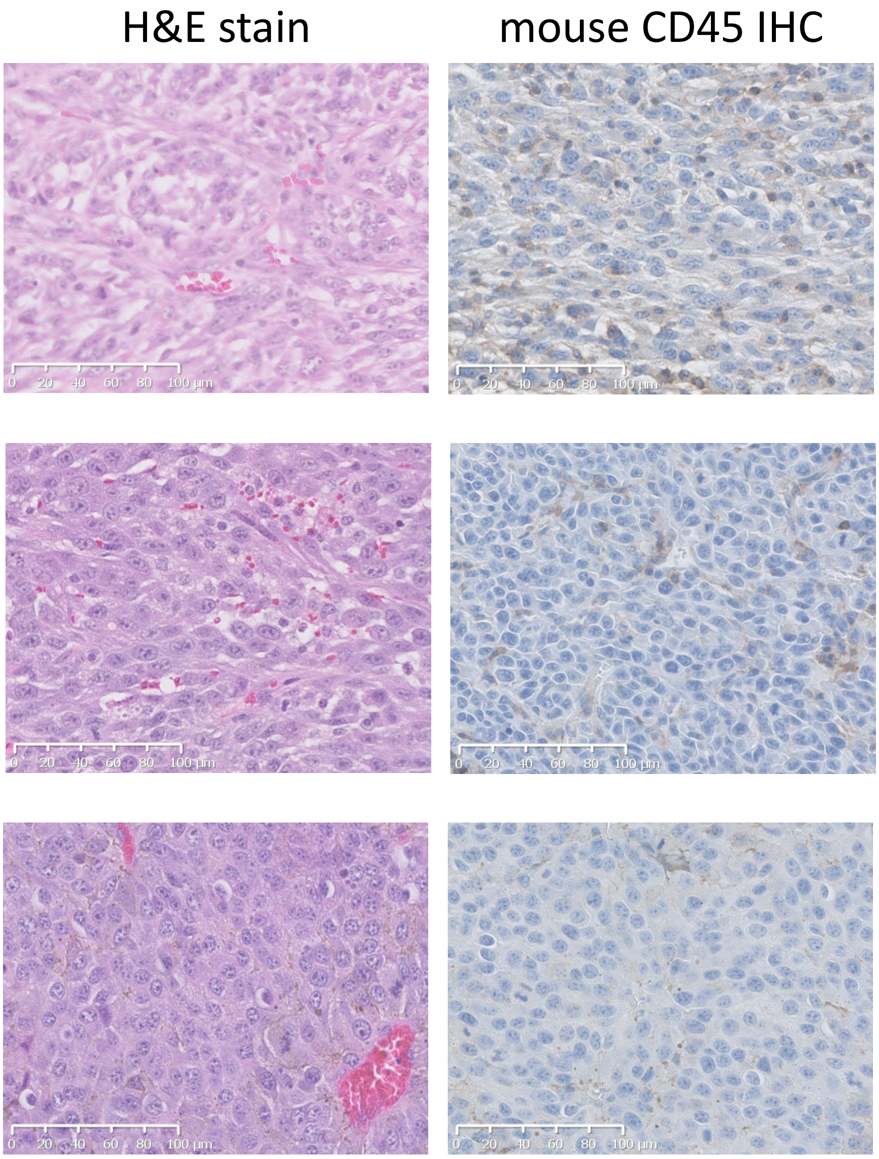

Supplemental Figure 1

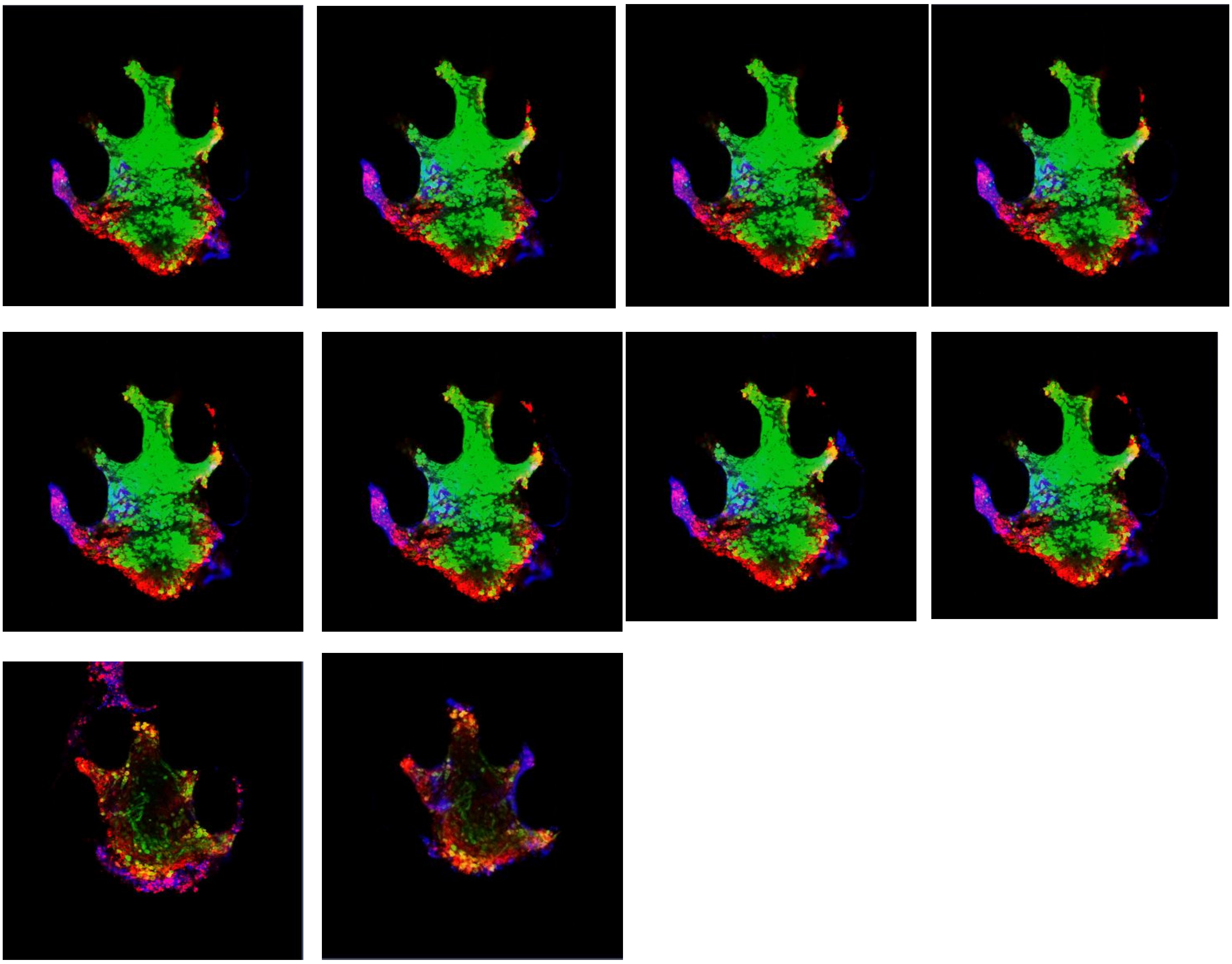

Time lapse high resolution confocal images taken at 3 hour intervals over the course of Day 2 for an MC38 tumor fragment exposed to flowing a-PD-1-treated TILs. Note the fading of green (live tissue) signal, increase in red (Annexin V dead cell signal), increase in blue signal (TILs, particularly at the left edge of the fragment over time. The last two images are at the end of Day 3 (24 hours after the previous image) and the end of Day 4 (an additional 24 hours later), showing continued reduction in green and increase in red signal.

Supplemental Figure 2

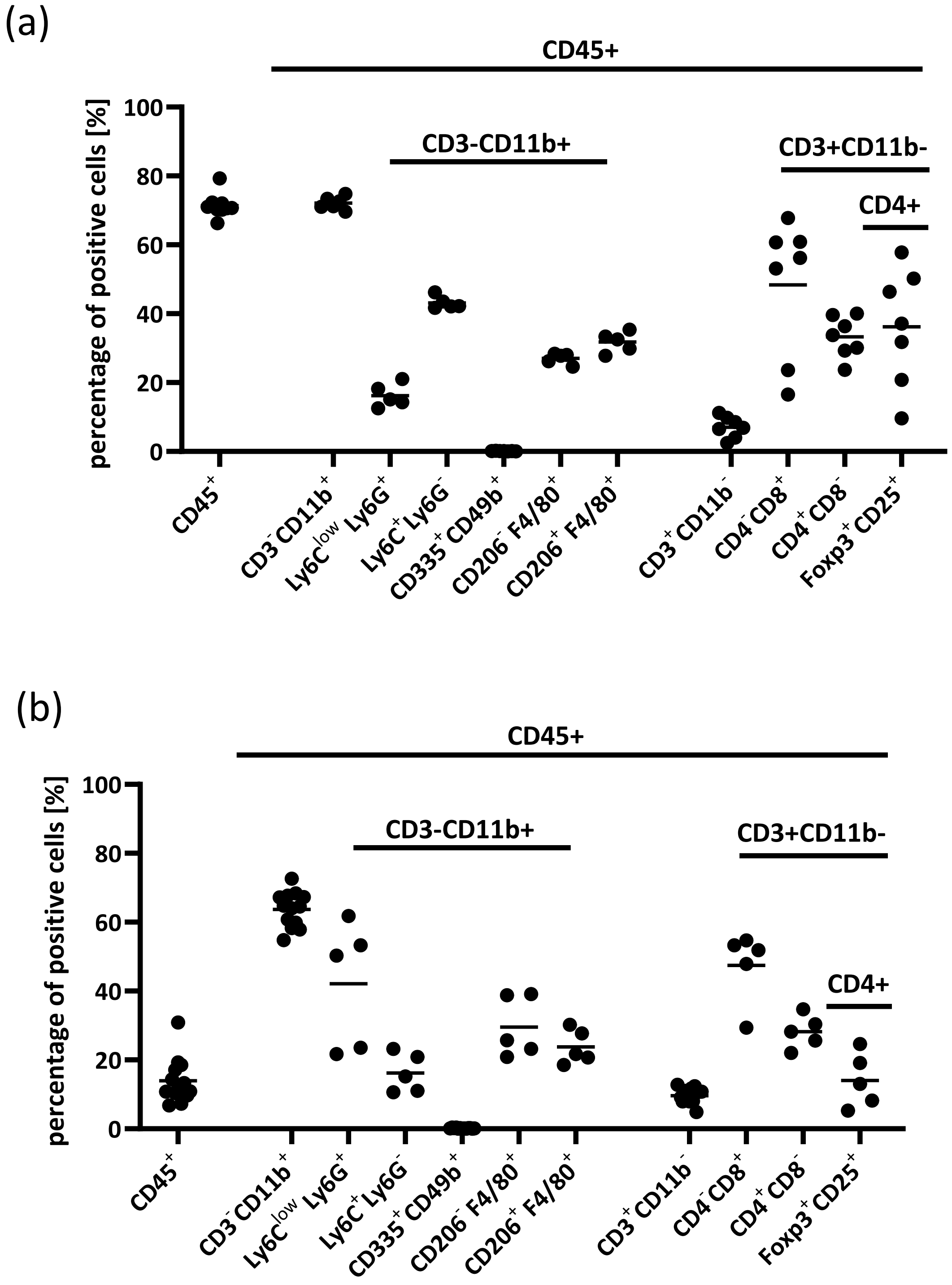

Flow cytometry analysis of tumor infiltrating lymphocytes in MC38 (a) and CT26 (b) tumors established subcutaneously in immune competent mice. MC38 displayed a higher TIL infiltration rate as CT26 (mean of 71% vs 14% CD45+ cells). The analyzed subtypes exhibited a similar distribution pattern in both tumor models.

Supplemental Figure 3
